# Simulation of the crosstalk between glucose and acetaminophen metabolism in a liver zonation model

**DOI:** 10.3389/fphar.2022.995597

**Published:** 2022-09-23

**Authors:** Kazuhiro Maeda, Shuta Hagimori, Masahiro Sugimoto, Yasuyuki Sakai, Masaki Nishikawa

**Affiliations:** ^1^ Department of Bioscience and Bioinformatics, Kyushu Institute of Technology, Iizuka, Fukuoka, Japan; ^2^ Department of Chemical System Engineering, University of Tokyo, Tokyo, Japan; ^3^ Institute of Medical Science, Tokyo Medical University, Tokyo, Japan; ^4^ Institute for Advanced Biosciences, Keio University, Yamagata, Japan

**Keywords:** zonation, acetaminophen, glucose, metabolism, hepatocytes, mathematical model

## Abstract

The liver metabolizes a variety of substances that sometimes interact and regulate each other. The modeling of a single cell or a single metabolic pathway does not represent the complexity of the organ, including metabolic zonation (heterogeneity of functions) along with liver sinusoids. Here, we integrated multiple metabolic pathways into a single numerical liver zonation model, including drug and glucose metabolism. The model simulated the time-course of metabolite concentrations by the combination of dynamic simulation and metabolic flux analysis and successfully reproduced metabolic zonation and localized hepatotoxicity induced by acetaminophen (APAP). Drug metabolism was affected by nutritional status as the glucuronidation reaction rate changed. Moreover, sensitivity analysis suggested that the reported metabolic characteristics of obese adults and healthy infants in glucose metabolism could be associated with the metabolic features of those in drug metabolism. High activities of phosphoenolpyruvate carboxykinase (PEPCK) and glucose-6-phosphate phosphatase in obese adults led to increased APAP oxidation by cytochrome P450 2E1. In contrast, the high activity of glycogen synthase and low activities of PEPCK and glycogen phosphorylase in healthy infants led to low glucuronidation and high sulfation rates of APAP. In summary, this model showed the effects of glucose metabolism on drug metabolism by integrating multiple pathways into a single liver metabolic zonation model.

## Introduction

The liver performs hundreds of vital functions such as metabolism of exogenous and endogenous compounds, bile production, and maintenance of blood levels of many substances, including albumin, glucose, amino acids, and vitamins (Trefts et al., 2017). Anatomically, the human liver contains approximately a million hepatic lobules with hexagonal cross-sections, and each lobule consists of a million hepatocytes and a thousand capillaries called sinusoids. The hepatic artery and the portal vein from the intestine merge at one end of the sinusoid in the periportal region of the lobule and flow into the central vein at the other end of the sinusoid in the pericentral region of the lobule (Abdel-Misih and Bloomston, 2010). Metabolic enzymes are expressed at different levels in hepatocytes along with the sinusoidal axis, which compartmentalizes hepatic functions (Halpern et al., 2017; Kietzmann, 2017; Halpern et al., 2018; Ben-Moshe et al., 2019). This spatial patterning of hepatic functions (hepatic zonation) presumably contributes to the efficient functional management of the whole organ since certain pathways antagonize each other.

Acetaminophen (APAP) is one of the most commonly used over-the-counter medications; however, its overdoses can lead to hepatotoxicity and acute liver failure (Sacks et al., 2018). APAP is intensively used as a model compound to study liver drug metabolism, and it is well known that hepatocytes in the pericentral region are initially damaged due to metabolic zonation (Anundi et al., 1993; Cunningham and Porat-Shliom, 2021). Multiple metabolic pathways are involved in APAP metabolism, and their mutual interaction and regulation add another layer of complexity to understanding the metabolic fate of APAP in the liver (Mazaleuskaya et al., 2015). In hepatocytes, APAP is eliminated through glucuronidation or sulfation or converted to the toxic intermediate metabolite *N*-acetyl-*p*-benzoquinone imine (NAPQI) by cytochrome P450 2E1 (CYP2E1), which is detoxified and eliminated through glutathione (GSH) conjugation. In adults, glucuronidation and sulfation play a major role in APAP metabolism, and approximately 5% of the substrate is converted into NAPQI (Court et al., 2001; Mutlib et al., 2006; Riches et al., 2009). Sulfation activity and GSH concentration are high in the periportal region, while glucuronidation, CYP2E1, and GSH conjugation are high in the pericentral region (Kietzmann, 2017). GSH is depleted in the pericentral region leading to site-specific accumulation of NAPQI, which covalently binds to cysteine (Cys) residues in proteins to form protein adducts, which cause hepatotoxicity (Yoon et al., 2016).

Glucuronidation accounts for the majority of APAP metabolic pathways in healthy adults (Jiang et al., 2013). Interestingly, uridine diphosphate (UDP)-glucuronic acid (UDP-GA), a substrate of glucuronide conjugation, is enzymatically produced from a glycogen metabolism metabolite constituting the glucose metabolism cascade (Adeva-Andany et al., 2016). This indicates that drug metabolism and glucose metabolism interact (Ghafoory et al., 2013; Dargue et al., 2020; Cunningham and Porat-Shliom, 2021). Glucose metabolism consisting of glycolysis, gluconeogenesis, and glycogen metabolism shows metabolic zonation in the liver (Kietzmann, 2017). Glucose uptake and glycolysis are promoted in the pericentral region, while gluconeogenesis and glucose delivery dominate in the periportal region.

The precise evaluation of complex human liver metabolism and toxicity may include *in silico* approaches as complementary or promising alternatives to existing *in vivo* and *in vitro* approaches. The first mathematical model for APAP metabolism (Reith et al., 2009) was followed by a multi-compartmental model (Ben-Shachar et al., 2012), a physiologically based pharmacokinetic model (Ben-Shachar et al., 2012; Jiang et al., 2013), and simplified models dealing with only major pathways (Remien et al., 2012; Reddyhoff et al., 2015). Recently, the zonation effects on APAP metabolism and the zone-specific hepatotoxicity were simulated in mathematical models (Smith et al., 2016; Franiatte et al., 2019; Kennedy et al., 2019; Means and Ho, 2019). Meanwhile, mathematical models incorporating zonal regulations of glycolysis, gluconeogenesis, and glycogen metabolisms were reported (Bulik et al., 2016; Berndt et al., 2018; Berndt and Holzhütter, 2018). However, most existing models considering metabolic zonation deal with drug metabolism or glucose metabolism individually. Thus, it is difficult to investigate crosstalk effects that impair the accuracy of evaluation under different clinical conditions.

In this study, we integrated metabolic zonation models of multiple pathways including drug and glucose metabolism into a single numerical human liver model. This model showed that changes in glucose metabolism enzyme activities affected APAP metabolism, and the crosstalk effects were dependent on liver zonation and nutritional status. These results showed the importance of incorporating multiple metabolic networks in a single zonation model.

## Materials and methods

### Model overview

**FIGURE 1:**
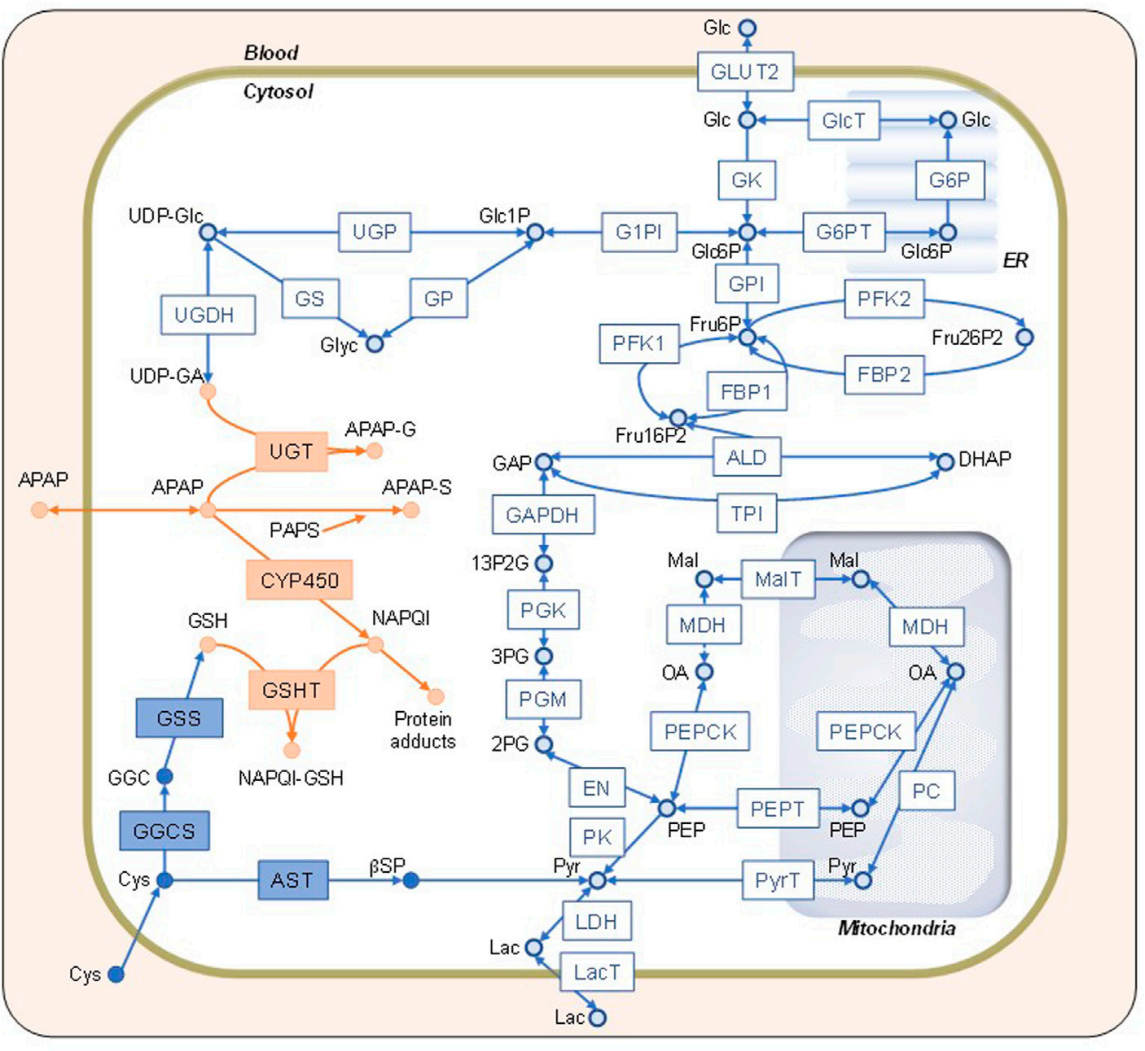
Network map of the constructed model. Schematic representation of the simulation model combining glucose (Glc), acetaminophen (APAP), and cysteine (Cys) metabolisms indicated by open blue, solid orange, and solid blue shapes, respectively. The definitions of the abbreviations are listed in the Supplementary Material. Glc, lactate, APAP, and Cys in the blood can be transported into the cytosol. The endoplasmic reticulum and mitochondria are shown on the background. The square boxes represent enzymes and transporters, whereas the circles represent substrates and metabolites.

We built a numerical liver zonation model by integrating glucose, APAP, and Cys metabolisms (Figure 1 and Supplementary Material for abbreviations). The glucose metabolism model consists of glycolysis, gluconeogenesis, and glycogen metabolism. Glucose metabolism rate equations were adopted and modified from previous studies (Bulik et al., 2016; Berndt et al., 2018; Berndt and Holzhütter, 2018). APAP metabolism utilized a previously described model (Means and Ho, 2019). Our model also contains Cys metabolism. We divided the liver into two zones for most cases and three zones for calculating glucose exchange rates and glycogen concentrations to compare with reported results (Berndt et al., 2018). The model consists of 34 variables (metabolites) and 47 reactions. We simulated metabolic zonation by changing enzyme activities depending on the zone that the cell belongs to: from the periportal-to the pericentral regions. For instance, the pericentral region has a greater expression of UTP-glucose-1-phosphate uridylyltransferase than the periportal region (Kietzmann, 2017). Thus, we used a higher Vmax value for UTP-glucose-1-phosphate uridylyltransferase in the pericentral cell. The model contains 13 zonation-dependent kinetic parameters (Supplementary Material). We assumed that the organism outside the liver was unchanged during simulations. The external glucose concentration and APAP administration rate were the boundary conditions, which we changed during simulations. We used the external glucose concentrations of 4 and 11 mM for the fasting and feeding states, respectively. We used the APAP administration rates of 0, 0.5, and 6 mM/h for no, moderate, and excessive administration, respectively. The liver zonation model was implemented in Python (ver.3.10, https://www.python.org) with NumPy library (ver. 1.21.0, https://numpy.org). The blood concentrations of glucose and APAP under different conditions, such as fasting, feeding, and moderate- and over-doses, were determined according to previous reports (Kennedy et al., 2019; Means and Ho, 2019; Lammers et al., 2020).

### Simulation method

Computer simulations of metabolic pathways often use dynamic simulation or metabolic flux analysis (MFA). The dynamic simulation reproduces the time-dependent transitions of metabolic concentrations but requires many parameters. For instance, the reversible Michaelis–Menten rate equation (commonly used for dynamic metabolic models) requires at least four parameters per enzyme reaction, leading to hundreds of parameters to construct a single metabolic model. Due to the limits of experimental data availability, optimizing many parameters is difficult. Meanwhile, MFA does not require any kinetic parameters. However, as it assumes that the entire system is at a steady state, a simple MFA cannot simulate any temporal changes in metabolite concentrations. Therefore, we used a hybrid approach in this study to compensate for the shortcomings of each method (Yugi et al., 2005). Briefly, dynamic simulation was applied to the rate-limiting reactions of the metabolic pathways, while MFA was utilized for the other reactions by assuming a pseudo-steady state at each simulation step.

In general, the dynamic behavior of a metabolic pathway is modeled by the following differential equation.

$$\frac{dx}{dt}=Sv$$ (1)

where ***x*** is the variable vector representing metabolite concentrations (n × 1), S is the stoichiometry matrix (n × m), and ***v*** is the reaction rate vector (m × 1), which is a function of ***x***. We divided the system into two modules: dynamic and static. The dynamic module contains key reactions that are bottlenecks of dynamic behaviors. The static module contains fast, non-bottleneck reactions. We divided ***v*** into reaction rates in the dynamic module (***v***_*d*_) and the static module (***v***_*s*_). We divided ***x*** into the variables only associated with dynamic module reactions (***x***_*d*_) and those with one or more static module reactions (***x***_*s*_). We divided S into three matrices: S_*d,d*_, S_*s,d*_, and S_*s,s*_. S_*d,d*_ is the stoichiometry matrix for the dynamic module variables and dynamic module reactions, S_*s,d*_ is for the static module variables and dynamic module reactions, and S_*s,s*_ is for the static module variables and static module reactions. Our model has 13 variables and 27 reactions in the dynamic module and 21 variables and 20 reactions in the static module. The incorporation of the new symbols allows the previous equation to be written as:

$$\left\{ \begin{array}{c} \frac{dx_{d}}{dt}=S_{d,d}v_{d}\\ \frac{dx_{s}}{dt}=S_{s,d}v_{d}+S_{s,s}v_{s} \end{array}\right.$$ (2)

S_*d,d*_, S_*s,d*_, and S_*s,s*_ values are given. ***v***_*d*_ is a set of kinetic rate equations such as mass actions and Michaelis-Menten rate equations, i.e., ***v***_*d*_ is a function of ***x***_*d*_ and ***x***_*s*_. Thus, the right-hand side of the upper equation can be calculated.

To calculate the right-hand side of the following equation, we assume the static module is at a steady state at each time step in a simulation. Thus, the following equation becomes:

$$0=S_{s,d}v_{d}+S_{s,s}v_{s}$$ (3)

In the over-determined case (including our model), there is no solution space for ***v***_*s*_ to satisfy the previous equation. Instead, we can obtain $v_{s}^{pseudo}$ , the most plausible alternative for ***v***_*s*,_ by the following equation:

$$v_{s}^{pseudo}={{-S}_{s,s}^{\#}S}_{s,d}v_{d}$$ (4)

where $S_{s,s}^{\#}$ is the Moore-Penrose pseudo-inverse of S_*s,s*_. $v_{s}^{pseudo}$ provides the least-squares estimate of the reaction rate distribution, which minimizes ${\left|S_{s,d}v_{d}+S_{s,s}v_{s}\right|}^{2}$ . This procedure equally distributes the error among the reaction rates of the static module (Yugi et al., 2005). Replacement of ***v***_*s*_ in the second equation with $v_{s}^{pseudo}$ enables time evolution simulation of **x**_*d*_ and **x**_*s*_.

## Results

### Comparison of the liver model with experimental data

**FIGURE 2:**
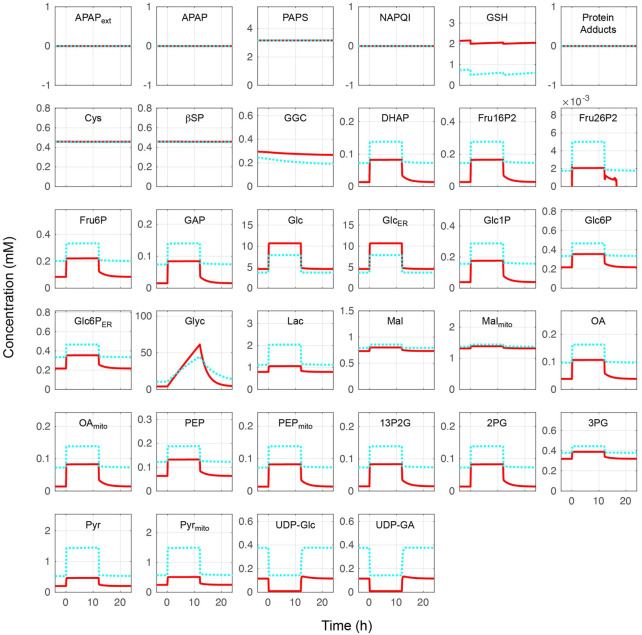
Metabolite concentrations without acetaminophen administration. Hepatocytes were in a feeding state from 0 to 12 h and in a fasting state for the rest of the simulation time. The external (blood) glucose levels were set to 4 and 11 mM for the fasting and feeding states, respectively. The solid red lines and blue dotted lines indicate the periportal and pericentral regions, respectively. The definitions of the abbreviations are listed in Supplementary Material.

**FIGURE 3:**
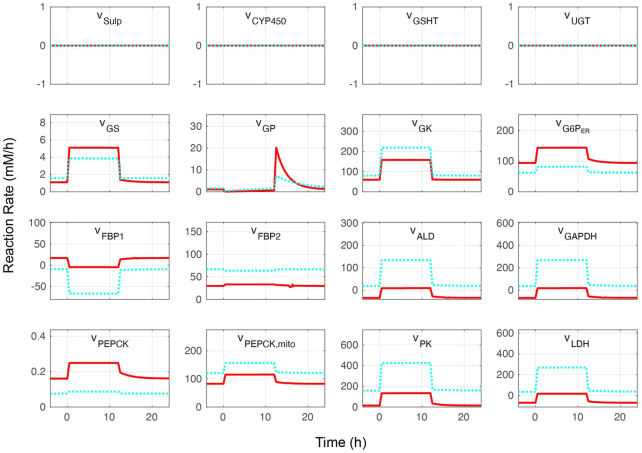
Metabolite reaction rates without acetaminophen administration. Hepatocytes were in a feeding state from 0 to 12 h and in a fasting state for the rest of the simulation time. The external (blood) glucose levels were set to 4 and 11 mM for the fasting and feeding states, respectively. The solid red lines and blue dotted lines indicate the periportal and pericentral regions, respectively. The definitions of the abbreviations are listed in Supplementary Material.

The whole combined model was initially performed at different blood glucose levels without APAP administration. Metabolite concentrations and reaction rates are shown in Figures 2, 3, respectively. As expected, the substrate- and metabolites concentrations related to APAP metabolisms such as APAP, NAPQI, and protein adducts were zero. Furthermore, the reaction rates of APAP sulfation (v_Sulf_), oxidation (v_CYP450_), glucuronidation (v_UGT_), and GSH conjugation (v_GSHT_) were zero. In contrast, metabolite concentrations and reaction rates related to glucose metabolism changed in response to the blood glucose level and reproduced zonation patterns with the values consistent with the liver (Halpern et al., 2017, 2018; Ben-Moshe et al., 2019: (Halpern et al., 2018; Ben-Moshe et al., 2019). The pericentral region had higher reaction rates of glucokinase (v_GK_), fructose-2,6-bisphosphatase (v_FBP2_), aldolase (v_ALD_), glyceraldehyde-3-phosphate dehydrogenase (v_GAPDH_), and pyruvate kinase (v_PK_) compared with the periportal region, and the positive lactate dehydrogenase reaction rate was indicative of glycolysis promotion and lactate (Lac) production (Miethke et al., 1985). The periportal region contained higher GSH concentration and greater reaction rates of fructose-1,6-bisphosphatase (v_FBP1_) and phosphoenolpyruvate carboxykinase in the cytosol (PEPCK, v_PEPCK_), and glucose-6-phosphate phosphatase in the endoplasmic reticulum (G6P, v_G6P_ER_) compared with the pericentral region (Katz et al., 1977; Ma et al., 2020). Lactate dehydrogenase works in the reverse direction in the fasting state, which favors gluconeogenesis. In contrast to cytosolic PEPCK, mitochondrial PEPCK contribution to glucose metabolism is not fully understood. Nevertheless, our model predicted that the reaction rate of mitochondrial PEPCK (v_PEPCKmito_) was higher in the pericentral region (Méndez-Lucas et al., 2013; Stark et al., 2014; Stark and Kibbey, 2014).

**FIGURE 4:**
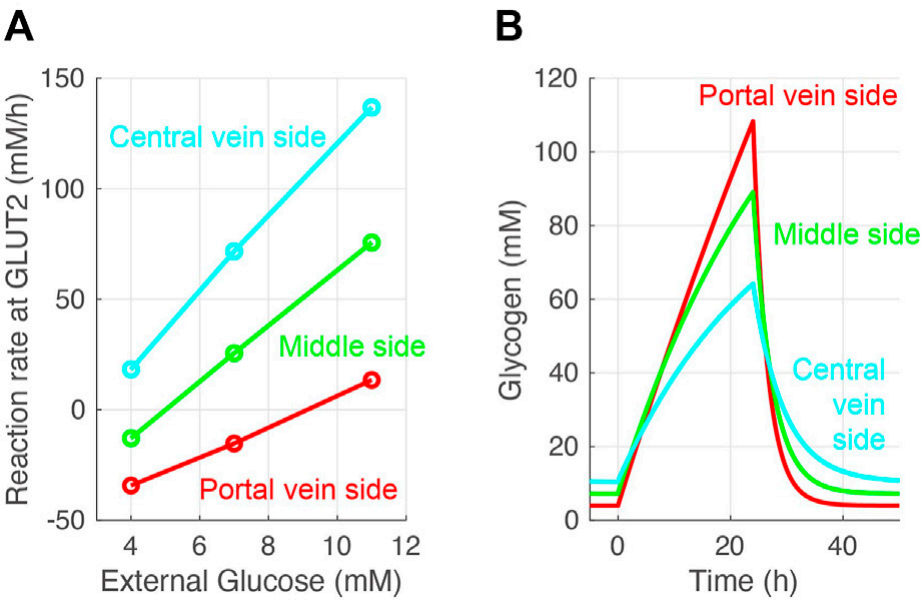
Validation of the constructed model. **(A)** Simulated glucose exchange rate by glucose transporter 2 in response to external (blood) glucose concentration. **(B)** Simulated glycogen concentrations over time. Hepatocytes were in a feeding state from 0 to 24 h and in a fasting state for the rest of the simulation time. The external (blood) glucose concentrations were set to 4 and 11 mM for the fasting and feeding states, respectively. The external lactose concentration was fixed at 1 mM in all simulations.

The glucose exchange rate by glucose transporter 2 in response to the external (blood) glucose concentration and the change of glycogen concentration over time was investigated to further validate the glucose metabolism model (Figure 4). The glucose transporter 2reaction rate increased with increased external glucose; in other words, cellular glucose consumption increased (Figure 4A). Pericentral cells consumed glucose faster than periportal cells. Periportal cells exported 4–9 mM glucose from external glucose concentrations. The nutritional-status-dependent glycogen concentration showed that its levels were lowest in periportal cells in the fasting state (Figure 4B). However, glycogen accumulated faster in periportal cells in the feeding state (from 0 to 24 h) and reached higher concentrations than that of intermediate and pericentral cells. These glycogen concentration transitions were congruous with the reaction rates of glycogen phosphorylase and glycogen synthase (GS) shown in Figure 3. These glucose metabolism results were consistent with existing observations and experimental data (Berndt et al., 2018).

**FIGURE 5:**
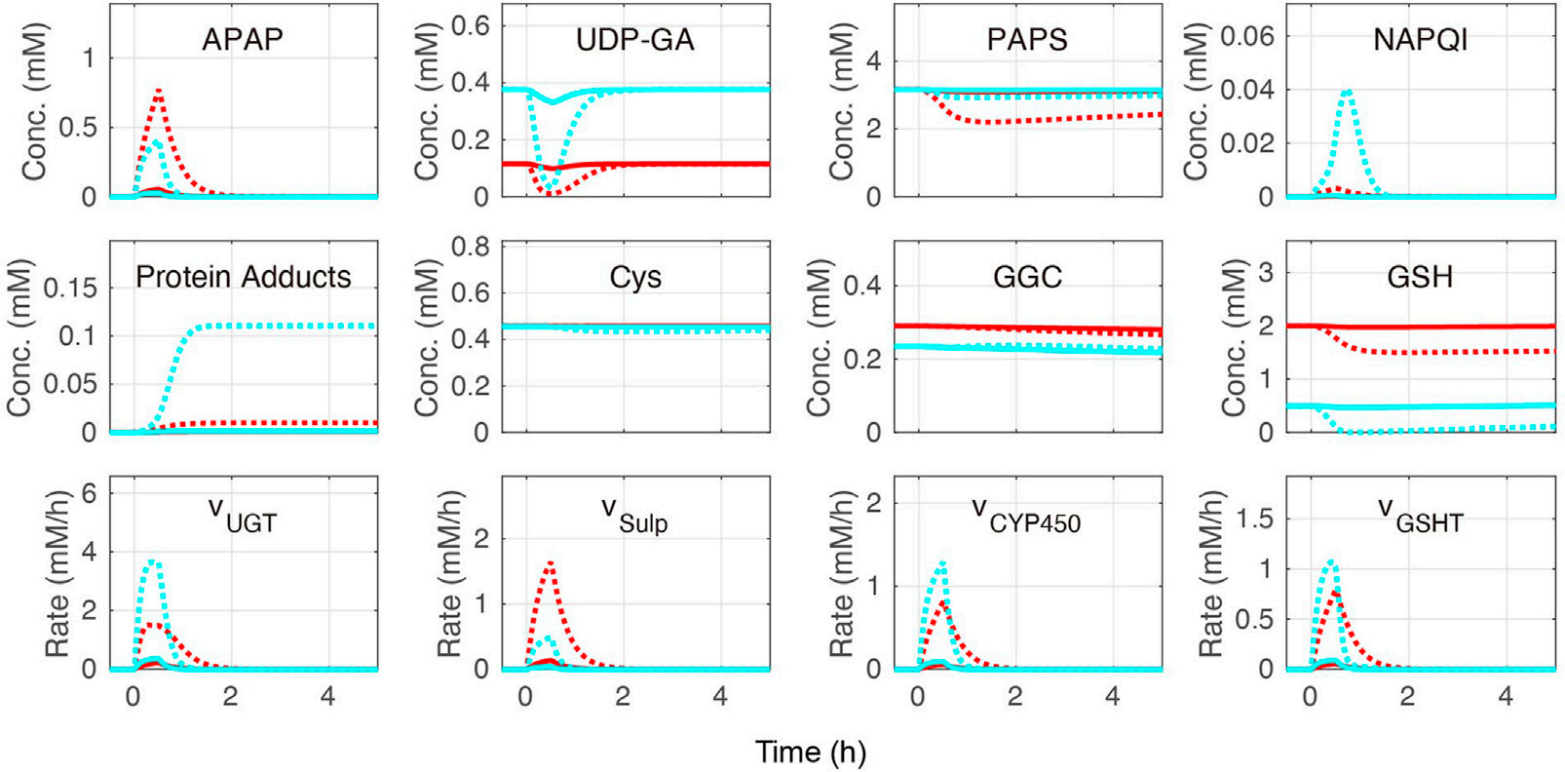
Simulation of acetaminophen (APAP) metabolism. APAP was administered for the initial 30 min of the simulation. The red and blue lines indicate the periportal and pericentral regions, respectively. The solid and dotted lines indicate moderate (0.5 mM/h) and excessive (6.0 mM/h) APAP administration, respectively. The definitions of the abbreviations are listed in Supplementary Material.

Cellular APAP concentration increased with its administration in the constructed model (Figure 5). After 2 h, APAP was eliminated by three APAP metabolic reactions: sulfation (v_Sulf_), oxidation (v_CYP450_), and glucuronidation (v_UGT_) (Figure 1). The rate of APAP sulfation (v_Sulf_) was higher in the periportal region than that in the pericentral region, while v_CYP450_ and v_UGT_ showed the opposite tendencies. A prominent increase of protein adducts leading to hepatotoxicity was observed in the pericentral region due to low GSH concentration and sulfation activity. Indeed, over-administration of APAP resulted in GSH depletion. Overall, our model was consistent with the existing observations in the pericentral region (Yoon et al., 2016; Ahn et al., 2019; Means and Ho, 2019).

Finally, the contribution of each APAP metabolic reaction was investigated (Table 1). According to a previous study (Mcgill and Jaeschke, 2013), APAP is eliminated mainly through glucuronidation (50%–70%), followed by sulfation (25%–35%) and oxidation (5%–15%). In our model, glucuronidation was also the main route of APAP elimination in most cases, and the range of contribution of each metabolic reaction was consistent with previous studies (Mcgill and Jaeschke, 2013) (Table 1). Sulfation and oxidation increased, while glucuronidation decreased following excessive administration of APAP (6.0 mM/h). Increasing protein adduct production in the pericentral region under these conditions is indicative of site-specific hepatotoxicity. Taken together, our liver metabolism model was validated through reproduced contributions of main APAP metabolic reactions and localized toxicity previously observed in the liver.

**TABLE 1:** Relative contribution of APAP metabolic pathways in healthy adults.

APAP administration (V_APAP_ext_) (mM/h)	Extracellular glucose (Glc_ext_) (mM)	Position	v_UGT_ (Glucuronidation) (%)	v_Sulf_ (Sulfation) (%)	v_CYP450_ (Oxidation)	
					GSHT (%)	Protein adduct production (%)
0.5	4	Periportal	53.8	32.5	13.4	0.2
0.5	4	Pericentral	73.7	7.5	18.4	0.4
0.5	11	Periportal	36.8	44.4	18.5	0.3
0.5	11	Pericentral	72.8	7.8	19.0	0.4
6.0	4	Periportal	47.1	35.2	17.3	0.3
6.0	4	Pericentral	70.4	8.2	17.6	3.7
6.0	11	Periportal	21.5	50.5	27.4	0.6
6.0	11	Pericentral	66.3	9.3	17.8	6.5

### Prediction

**FIGURE 6:**
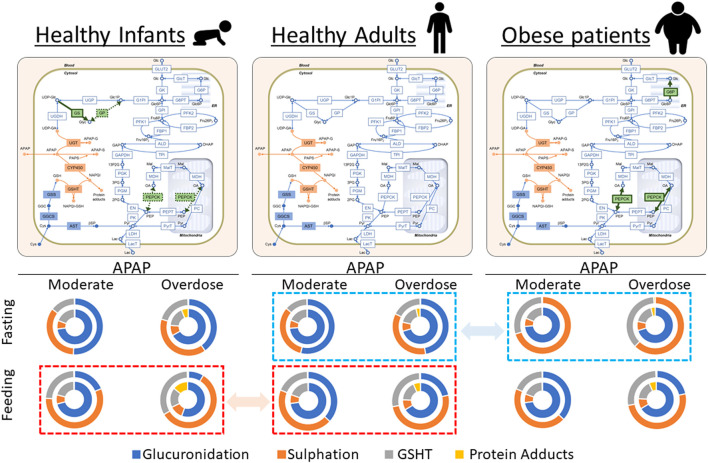
Visual representation of the results in Tables 1, 2, and 3. Acetaminophen (APAP) was administered for the initial 30 min at 0.5 mM/h (moderate dose) or 6.0 mM/h (overdose) under either 4 mM (fasting) or 11 mM (feeding) of blood glucose. The reaction rates of APAP metabolic pathways were integrated up to 5 h, and the results are shown as relative contributions to APAP metabolism in pie charts. In each chart, the outer and inner circles represent the results in the periportal and pericentral regions, respectively. The activities of phosphoenolpyruvate carboxykinase (PEPCK) and glucose-6-phosphate phosphatase (G6P) increased in obese adults compared with healthy adults. Furthermore, the glycogen synthase activity increased, and PEPCK and glycogen phosphorylase activities decreased in healthy infants compared with healthy adults. The modified enzymatic activities are highlighted in green on the network maps. Dashed squares (red to red and blue to blue) highlight the prominent differences in relative contributions of APAP metabolic pathways among three model cases.

It is widely known that obese and healthy individuals have different enzyme activities. For example, PEPCK and G6P activities are increased in obese individuals (Ropelle et al., 2009; Wang et al., 2011). Thus, we increased the enzyme activities (V_max_) of PEPCK and G6P by 50% in our model and investigated the effects on APAP metabolism. Interestingly, increased PEPCK and G6P activities influenced APAP metabolism in only two cases out of eight (bold letters in Table 2; Figure 6): both cases were in the periportal region in the fasting state. In both cases, glucose-1-phosphate, UDP-glucose, and UDP-GA (metabolites connecting glycolysis and APAP metabolism) were depleted. UDP-GA is a substrate of glucuronidation; therefore, APAP could not be metabolized by this pathway. Decreased glucuronidation in the periportal region led to increased sulfation and oxidation by CYP2E1, suggesting the potential risk of increased hepatotoxicity in obese individuals.

**TABLE 2:** Relative contribution of APAP metabolic pathways in obese patients.

APAP administration (V_APAP_ext_) (mM/h)	Extracellular glucose (Glc_ext_) (mM)	Position	v_UGT_ (Glucuronidation) (%)	v_Sulf_ (Sulfation) (%)	v_CYP450_ (Oxidation)	
					GSHT (%)	Protein adduct production (%)
**0.5**	**4**	**Periportal**	**0**	**70.1**	**29.4**	**0.5**
0.5	4	Pericentral	73.7	7.5	18.4	0.4
0.5	11	Periportal	36.9	44.3	18.4	0.3
0.5	11	Pericentral	72.8	7.8	19.0	0.4
**6.0**	**4**	**Periportal**	**0**	**62.5**	**36.7**	**0.9**
6.0	4	Pericentral	70.3	8.3	17.7	3.8
6.0	11	Periportal	21.6	50.5	27.4	0.6
6.0	11	Pericentral	66.4	9.3	17.8	6.5

Infants have low PEPCK and glycogen phosphorylase activities and high GS activity compared with adults (Mitanchez, 2007). Sulfation is the major conjugation pathway in drug metabolism in children, while glucuronidation is dominant in adults (Jiang et al., 2013). We simulated the infant glucose metabolism by increasing the enzyme activities of PEPCK and glycogen phosphorylase and decreasing the GS activity using our liver metabolism model to investigate the possible mechanistic interconnection behind these two observations (Figure 6). There was decreased glucuronidation and increased sulfation in all cases in infants compared with adults (Tables 1 and 3). The impact was the most prominent in the state of feeding (Figure 6). For example, adults mainly used glucuronidation (66.3%) for APAP metabolism in the pericentral region with excessive APAP administration (Table 1). In contrast, glucuronidation decreased to 55.6% of the APAP metabolism in infants, while both sulfation and oxidation increased as compensation. This resulted in a drastic increase in protein adducts from 6.5% to 14.2%. These results showed that infants were susceptible to APAP-induced hepatotoxicity more than adults because of the reduced glucuronidation activity and increased oxidation.

**TABLE 3:** Relative contribution of APAP metabolic pathways in healthy infants.

APAP administration (V_APAP_ext_) (mM/h)	Extracellular glucose (Glc_ext_) (mM)	Position	v_UGT_ (Glucuronidation) (%)	v_Sulf_ (Sulfation) (%)	v_CYP450_ (Oxidation)	
					GSHT (%)	Protein adduct production (%)
0.5	4	Periportal	50.8	34.7	14.3	0.2
0.5	4	Pericentral	73.0	7.7	18.8	0.4
**0.5**	**11**	**Periportal**	**18.8**	**57.0**	**23.8**	**0.4**
0.5	11	Pericentral	70.9	8.3	20.3	0.5
6.0	4	Periportal	40.2	39.5	19.9	0.4
6.0	4	Pericentral	67.0	9.2	17.8	6.1
**6.0**	**11**	**Periportal**	**8.7**	**57.7**	**32.8**	**0.7**
**6.0**	**11**	**Pericentral**	**55.6**	**12.2**	**18.1**	**14.2**

## Discussion

In this study, we integrated multiple metabolic pathways including drug and glucose metabolism into a single numerical liver zonation model and validated it with the results and observations of previous reports in a semi-quantitative manner. Then, we investigated the possible crosstalk between those pathways. The model showed the effects of glucose metabolism on drug metabolism in different contexts, which indicated the importance of integrating multiple metabolic pathways.

The glucose metabolism model consisting of glycolysis, gluconeogenesis, and glycogen metabolism was determined by adopting and modifying previous rate equations (Bulik et al., 2016; Berndt et al., 2018; Berndt and Holzhütter, 2018). APAP metabolism was determined by modifying a previous model (Means and Ho, 2019). Our model also contained Cys metabolism. The combined model was validated by initially testing it without APAP administration at different blood glucose levels representing fasting and feeding states. Overall, the glucose metabolism metabolite concentrations and reaction rates in our model were consistent with existing observations of liver zonation (Figures 2–4) (Halpern et al., 2017; Halpern et al., 2018; Ben-Moshe et al., 2019). Drug metabolism in the constructed model was validated by monitoring metabolite concentrations and reaction rates of APAP metabolic pathways under moderate- and excessive APAP administration in fasting and feeding states (Figure 5). Besides, the relative contribution of each APAP metabolic reaction was investigated (Table 1). High reaction rates and contributions of glucuronidation and oxidation were observed in the pericentral region, while sulfation was promoted in the periportal region. Zonal characteristics and the contribution ranges of each metabolic reaction were similar to a previous study (Mcgill and Jaeschke, 2013). Besides, a prominent increase of protein adducts leading to hepatotoxicity was observed in the pericentral region due to GSH depletion. Altogether, our model reproduced the main contribution of APAP metabolic reactions and localized toxicity observed in the liver (Yoon et al., 2016; Ahn et al., 2019; Means and Ho, 2019).

The model showed that drug metabolism was affected by nutritional status (Table 1). The nutritional status changed the concentrations of UDP-glucose and UDP-GA and the GS reaction rate (Figures 2, 3). The low concentration of UDP-GA in the feeding state seemed to slow down glucuronidation. Decreased glucuronidation in the feeding state under moderate APAP administration was compensated by sulfation and oxidation followed by GSH conjugation and did not lead to increased protein adducts. Sulfation and GSH conjugation were promoted during excessive APAP administration; however, protein adducts increased in the feeding state, especially in the pericentral region. In general, drug administration during the feeding state is considered safer than that during the fasting state, mostly because the rate of drug administration through the digestive system decreases during feeding. However, our model suggested that the feeding state could have adverse effects by altering liver metabolism and result in higher hepatotoxicity, provided that other things are equal. This result may be slightly controversial; however, it at least suggests the necessity of careful interpretation of *in vivo* observation and demonstrates the advantage of simultaneously considering multiple metabolic pathways.

Our integration model was also useful for proposing hypothetical insights into the mechanisms of metabolic features involving glucose- and drug metabolism. Increased activities of PEPCK and G6P in glucose metabolism are reported in obese patients (Ropelle et al., 2009; Wang et al., 2011). Meanwhile, CYP2E1-mediated oxidation and sulfation in drug metabolism are higher than that of non-obese individuals (Brill et al., 2012; Van Rongen et al., 2016). We found that the contributions of CYP2E1-mediated oxidation and sulfation became higher in obese patients than that in healthy adults following increasing PEPCK- and G6P activities to reproduce glucose metabolism (Tables 1 and 2; Figure 6). This suggested that the increased activities of PEPCK and G6P could at least partly account for increased CYP2E1-mediated oxidation and sulfation in obese patients. However, there was a discrepancy in the contribution of glucuronidation between our model and reported observations (Brill et al., 2012; Van Rongen et al., 2016). Therefore, the model could be improved by considering activity changes in drug metabolism in obese patients, and the amount of glycogen stored in the liver may need to be adjusted for obese patients. On the contrary, these results suggest the difficulty of mechanism-based interpretations of *in vivo* observations and the complementarity of *in vivo* and *in silico* approaches. We also investigated the case of healthy infants in our model by adjusting some of the glucose metabolism enzymatic activities to reproduce healthy infant characteristics according to previous reports (Mitanchez, 2007). The adjustments affected drug metabolism, which successfully recapitulated the difference in relative contributions of glucuronidation and sulfation between adults and infants (Jiang et al., 2013). Although enzymatic activities in drug metabolism can have a larger impact on drug metabolism, our model suggested that the crosstalk between glucose and drug metabolism should not be neglected for the precise assessment of drug kinetics.

The study has some limitations. The model successfully reproduced periportal and pericentral characteristics of metabolic zonation regarding many metabolites and reaction rates in APAP and glucose metabolisms. Nonetheless, such validations were performed by comparing the present results with the results and observations of previous studies in a semi-quantitative manner, and we performed these validations using a limited number of conditions regarding glucose and APAP blood concentrations. The model contains only 13 zonation-dependent kinetic parameters (Supplementary Material), and metabolic zonation was simulated by manually changing enzyme activities depending on the zone that the cell belongs to, either the periportal or pericentral region. Conversely, the actual zonation is gradual and continuous along the sinusoidal axis without any histological borders and involves crosstalks of various metabolisms (Panday et al., 2022). This model also lacked the dynamic interaction of hepatocytes between the periportal and the pericentral regions through sinusoidal blood flow (Berndt and Holzhütter, 2018; Kennedy et al., 2019) and omitted the regulatory effects of oxygen and growth factors that are critical for maintaining metabolic zonation (Schmierer et al., 2010; Kietzmann, 2017; Scheidecker et al., 2020). Furthermore, there was a discrepancy between our model and the reported *in vivo* observations in drug metabolism in obese patients. To overcome these limitations, the modification of boundary conditions and parameters in drug metabolism based on actual measurements may lead to better prediction. The contribution of fatty acid metabolism is another challenge, especially for the better reproduction of energy metabolism in obese patients (Aubert et al., 2011; Berndt et al., 2021). To understand the regulatory mechanisms, the effects of hypoxia-inducible factor and coenzymes such as ATP may need to be considered as dependent variables of blood oxygen levels (Ainscow and Brand, 1999; Maher et al., 2007; Rankin et al., 2009). Considering future perspectives, the model may contribute to drug development and personalized medicine. The incorporation of multiscale regulatory networks ranging from gene expression to metabolisms will facilitate the complementary use of such models in *in vivo* and *in vitro* experiments, contributing to a better understanding and prediction of complex biological systems (Brunak et al., 2020; Waltemath et al., 2020).

To conclude, we integrated multiple metabolic zonation models into a single numerical human liver model and validated it with the results and observations of previous reports in a semi-quantitative manner. This model showed that changes in the enzyme activities of glucose metabolism affected APAP metabolism, and the crosstalk effects were dependent on liver zonation and nutritional status. These results showed the importance of incorporating multiple metabolic networks in a single zonation model.

## Data Availability

The raw data supporting the conclusions of this article will be made available by the authors, without undue reservation.

## Abbreviation

APAP, Acetaminophen; CYP2E1, cytochrome P450 2E1; Cys, cysteine; G6P, glucose-6-phosphate phosphatase; GS, glycogen synthase; GSH, glutathione; MFA, metabolic flux analysis; NAPQI, *N*-acetyl-*p*-benzoquinone imine; PEPCK, phosphoenolpyruvate carboxykinase; UDP, uridine diphosphate; UDP-GA, UDP-glucuronic acid.
