# Supplementary material for: Simulation of the crosstalk between glucose and acetaminophen metabolism in a liver zonation model
Source: Front Pharmacol. 2022 Sep 23;13:995597. doi: 10.3389/fphar.2022.995597 (PMC9537759; doi:10.3389/fphar.2022.995597)
Supplement: Supplementary file 1 [file DataSheet1.pdf]

Supplementary Material

1 Abbreviations of substrates and proteins in Figure 1

In the Type column, M/S, E, and T indicate Metabolite or Substrate, Enzyme, and Transporter, respectively.

Abbreviations	Full Name	Type
13P2G	1,3-bisphosphoglycerate	M/S
2PG	2-phosphoglycerate	M/S
3PG	3-phosphoglycerate	M/S
ALD	Aldolase	E
APAP	acetaminophen (N-acetyl-para-aminophenol, paracetamol)	M/S
APAP-G	acetaminophen glucuronide	M/S
APAP-S	acetaminophen sulfate	M/S
AST	aspartate aminotransferase	E
CYP450	cytochrome P450	E
Cys	cysteine	M/S
DHAP	dihydroxyacetonephosphate	M/S
EN	enolase	E
FBP1	fructose-1,6-bisphosphatase	E

FBP2	fructose-2,6-bisphosphatase	E
Fru16P2	fructose-1,6-bisphosphate	M/S
Fru26P2	fructose-2,6-bisphosphate	M/S
Fru6P	fructose-6-phosphate	M/S
G6P	glucose-6-phosphate phosphatase	E
G6PT	glucose-6-phosphate transporter	T
GAP	glyceraldehydephosphate	M/S
GAPDH	glyceraldehyde 3-phosphate dehydrogenase	E
GGC	gamma-glutamylcysteine	M/S
GGCS	gamma-glutamyl-cysteine synthetase	E
GK	glucokinase	E
GLUT2	glucose transporter 2	T
GP	glycogen phosphorylase	E
GPI	glucose-6-phosphate isomerase	E
GS	glycogen synthase	E
GSH	glutathione	M/S
GSHT	glutathione S-transferase	E
GSS	glutathione synthetase	E
Glc	glucose	M/S
Glc1P	glucose-1-phosphate	M/S

Glc6P	glucose-6-phosphate	M/S
GlcT	glucose transporter	T
Glyc	glycogen	M/S
LDH	lactate dehydrogenase	E
Lac	lactate	M/S
LacT	lactate transporter	T
MDH	malate dehydrogenase	E
Mal	malate	M/S
MalT	malate transporter	T
NAPQI	N-acetyl-p-benzoquinone imine	M/S
NAPQI-GSH	NAPQI-glutathione conjugate	M/S
NDK	nucleoside-diphosphate kinase	E
OA	oxaloacetate	M/S
P1PI	glucose-1-phosphate isomerase	E
PAPS	3'-phosphoadenosine-5'-phosphosulfate	E
PC	pyruvate carboxylase	E
PEP	phosphoenolpyruvate	M/S
PEPCK	phosphoenolpyruvate carboxykinase	E
PEPT	phosphoenolpyruvate transporter	T
PFK1	phosphofructokinase 1	E

PFK2	phosphofructokinase 2	E
PGK	phosphoglycerate kinase	E
PGM	phosphoglycerate mutase	E
PK	pyruvate kinase	E
Protein adducts	covalent binding of NAPQI to cysteine residues in proteins	M/S
Pyr	pyruvate	M/S
PyrT	pyruvate transporter	T
TPI	triosephosphate isomerase	E
UDGH	UDP-glucose 6-dehydrogenase (referred as UG6D in the source code)	E
UDP	uridine diphosphate	M/S
UDP-GA	glucuronic acid	M/S
UDP-Glc	UDP-glucose	M/S
UGP	UDP-glucose pyrophosphorylase	E
UGT	glycuronosyl transferase	E
β SP	beta-sulfinylpyruvate	M/S

2 Initial concentrations

Name	Initial concentration (mM)

$APAP_{ext}$	0
APAP	0
PAPS	3.16
NAPQI	0
GSH	3.28
Protein Adducts	0
Cys	0
βSP	0
GGC	0.38
DHAP	4.19×10^{-12}
Fru16P2	8.08×10^{-4}
Fru26P2	5.40×10^{-4}
Fru6P	5.70×10^{-2}
GAP	1.91×10^{-3}
Glc	4.92
Glc_{ER}	4.92
Glc1P	1.17×10^{-2}
Glc6P	0.190
$Glc6P_{ER}$	0.190
Glyc	17.6

Lac	0.679
Mal	0.720
Mal_{mito}	1.30
OA	2.44×10^{-2}
OA_{mito}	4.65×10^{-4}
PEP	4.99×10^{-2}
PEP_{mito}	1.11×10^{-4}
13P2G	1.09×10^{-3}
2PG	2.94×10^{-4}
3PG	0.306
Pyr	8.52×10^{-2}
Pyr_{mito}	0.132
UDP-Glc	4.00×10^{-2}
UDP-GA	1.30×10^{-2}

3 Fixed molecular concentrations

Name	Concentration (mM)
Glc_{ext}	4.00 for the fasting state

	11.0 for the feeding state
Lac_{ext}	1.00
ADP	0.500
ADP_{mito}	7.50
AMP	0.160
ATP	3.25
ATP_{mito}	17.5
CO2	5.00
$CO2_{mito}$	5.00
P	5.00
P_{mito}	8.00
PP	0.008
GTP	0.693
GTP_{mito}	0.240
GDP	0.107
GDP_{mito}	0.560
UDP	1.15

4 Zonation-dependent kinetic parameters

	Unit	Periportal	Intermediate	Pericentral
k_S	$mM^{-1}h^{-1}$	0.8	0.6	0.4
k_{450}	h^{-1}	1.05	2.10	3.15
k_{GSH}	$mM^{-1}h^{-1}$	1.39×10^2	2.79×10^2	4.18×10^2
k_G	mM/h	18.3	33.5	51.1
$V_{max,GGCS}$	mM/h	2.0	1.5	1.0
$V_{max,GSS}$	mM/h	1.5	1.0	0.5
$V_{max,GK}$	mM/h	3.18×10^3	4.77×10^3	6.36×10^3
$V_{max,G6PER}$	mM/h	8.91×10^2	6.48×10^2	4.05×10^2
$V_{max,PEPCK}$	mM/h	7.75×10^2	5.11×10^2	2.43×10^2
$V_{max,PFK2}$	mM/h	6.64×10	9.97×10	1.33×10^2
$V_{max,FBP2}$	mM/h	7.88×10^2	1.18×10^3	1.57×10^3
$V_{max,PK}$	mM/h	6.13×10^3	7.45×10^3	8.77×10^3
$V_{max,UGP}$	mM/h	7.78×10^2	1.17×10^3	1.56×10^3

5 Zonation- and extracellular glucose-dependent kinetic parameters

Units are mM/h.

$$V_{max,GP} = \begin{cases} 1.93 \times 10^2 \cdot \left(1 + 39 \dfrac{([Glc_{ext}] - 4)^4}{([Glc_{ext}] - 4)^4 + 3^4}\right)^{-1} & (Periportal) \\ 1.40 \times 10^2 \cdot \left(1 + 9 \dfrac{([Glc_{ext}] - 4)^4}{([Glc_{ext}] - 4)^4 + 3^4}\right)^{-1} & (Intermediate) \\ 8.69 \times 10^1 \cdot \left(1 + 4 \dfrac{([Glc_{ext}] - 4)^4}{([Glc_{ext}] - 4)^4 + 3^4}\right)^{-1} & (Pericentral) \end{cases}$$

$$V_{max,GS} = \begin{cases} 1.02 \times 10^2 \cdot \left(1 + 39 \dfrac{([Glc_{ext}] - 4)^4}{([Glc_{ext}] - 4)^4 + 3^4}\right) & (Periportal) \\ 6.80 \times 10^1 \cdot \left(1 + 9 \dfrac{([Glc_{ext}] - 4)^4}{([Glc_{ext}] - 4)^4 + 3^4}\right) & (Intermediate) \\ 3.45 \times 10^1 \cdot \left(1 + 4 \dfrac{([Glc_{ext}] - 4)^4}{([Glc_{ext}] - 4)^4 + 3^4}\right) & (Pericentral) \end{cases}$$

6 Balance Equations

Units are mM/h.

$$\frac{d[APAP_{ext}]}{dt} = v_{APAP_{ext}} - v_{APAP_{tocell}} + v_{APAP_{toblood}}$$

$$\frac{d[APAP]}{dt} = -v_{Sulp} - v_{Gluc} - v_{Oxid} + v_{APAP_{tocell}} - v_{APAP_{toblood}}$$

$$\frac{d[PAPS]}{dt} = -v_{Sulp} + v_{PAPS}$$

$$\frac{d[NAPQI]}{dt} = v_{Oxid} - v_{GSHB} - v_{DPB}$$

$$\frac{d[GSH]}{dt} = -v_{GSHB} + v_{GSHS}$$

$$\frac{d[Protein\ Adducts]}{dt} = v_{DPB}$$

$$\frac{d[Cys]}{dt} = v_{Cys} - v_{GGCS} - v_{PLP}$$

$$\frac{d[\beta SP]}{dt} = v_{AST} - v_{Pyr}$$

$$\frac{d[GGC]}{dt} = v_{GGCS} - v_{GSS}$$

$$\frac{d[DHAP]}{dt} = v_{ALD} - v_{TPI}$$

$$\frac{d[Fru16P2]}{dt} = v_{PFK1} - v_{FBP1} - v_{ALD}$$

$$\frac{d[Fru26P2]}{dt} = v_{PFK2} - v_{FBP2}$$

$$\frac{d[Fru6P]}{dt} = v_{GPI} - v_{PFK1} - v_{PFK2} + v_{FBP1} + v_{FBP2}$$

$$\frac{d[GAP]}{dt} = v_{ALD} + v_{TPI} - v_{GAPDH}$$

$$\frac{d[Glc]}{dt} = v_{GLUT2} - v_{GK} + v_{GlcT_{ER}}$$

$$\frac{d[Glc_{ER}]}{dt} = v_{G6P_{ER}} - v_{GlcT_{ER}}$$

$$\frac{d[Glc1P]}{dt} = v_{GP} - v_{G1PI} - v_{UGP}$$

$$\frac{d[Glc6P]}{dt} = v_{GK} + v_{G6PT_{ER}} - v_{GPI} + v_{G1PI}$$

$$\frac{d[Glc6P_{ER}]}{dt} = -v_{G6P_{ER}} - v_{G6PT_{ER}}$$

$$\frac{d[Glyc]}{dt} = v_{GS} - v_{GP}$$

$$\frac{d[Lac]}{dt} = v_{LacT} + v_{LDH}$$

$$\frac{d[Mal]}{dt} = v_{MalT} - v_{MDH} + v_{PyrMalT}$$

$$\frac{d[Mal_{mito}]}{dt} = -v_{MalT} - v_{MDH_{mito}} - v_{PyrMalT}$$

$$\frac{d[OA]}{dt} = v_{MDH} - v_{PEPCK}$$

$$\frac{d[OA_{mito}]}{dt} = v_{PC} - v_{PEPCK_{mito}} + v_{MDH_{mito}}$$

$$\frac{d[PEP]}{dt} = v_{EN} - v_{PK} + v_{PEPCK} - v_{PEPT}$$

$$\frac{d[PEP_{mito}]}{dt} = v_{PEPCK_{mito}} + v_{PEPT}$$

$$\frac{d[13P2G]}{dt} = v_{GAPDH} - v_{PGK}$$

$$\frac{d[2PG]}{dt} = v_{PGM} - v_{EN}$$

$$\frac{d[3PG]}{dt} = v_{PGK} - v_{PGM}$$

$$\frac{d[Pyr]}{dt} = v_{PK} - v_{LDH} - v_{PyrT} - v_{PyrMalT} + v_{Pyr}$$

$$\frac{d[Pyr_{mito}]}{dt} = v_{PyrT} - v_{PC} + v_{PyrMalT}$$

$$\frac{d[UDP-Glc]}{dt} = v_{UGP} - v_{GS} - v_{UG6D}$$

$$\frac{d[UDP-GA]}{dt} = v_{UG6D} - v_{Gluc}$$

7 Rate Equations

Reaction rates (fluxes) are in mM/h. Reaction rates with 'FAST' are calculated by the flux balance analysis (see Main Text).

$$v_{Sulp} = k_S[PAPS][APAP]$$

$$v_{Oxid} = k_{450}[APAP]$$

$$v_{GSHB} = k_{GSH}[NAPQI][GSH]$$

$$v_{DPB} = 4.58 \cdot [NAPQI]$$

$$v_{Gluc} = \frac{k_G[APAP]}{[APAP] + 4} \frac{[UDP - GA]}{[UDP - GA] + 0.01}$$

$$v_{PAPS} = 0.264 - 0.0833[PAPS]$$

$v_{APAP_{ext}} = 0$ for the normal, 0.5 for the normal administration, 6.0 for the over administration

$$v_{APAP_{tocell}} = 50 \cdot [APAP_{ext}]$$

$$v_{APAP_{toblood}} = 50 \cdot [APAP]$$

$$v_{Cys} = 0.5 - 0.0833[Cys]$$
$$v_{AST} = 1.0 \cdot [Cys]$$
$$v_{Pyr} = 1.0 \cdot [B\_SP]$$

$$v_{GGCS} = \frac{V_{max,GGCS}\left(\frac{0.1}{0.1 + [GSH]}\right)\left([Cys] - \frac{[GGC]}{2}\right)}{1 + \frac{[Cys]}{0.1} + \frac{[GGC]}{0.1}}$$

$$v_{GSS} = \frac{V_{max,GSS}\left([GGC] - \frac{[GSH]}{10}\right)}{1 + \frac{[GGC]}{0.1} + \frac{[GSH]}{0.1}}$$

$$v_{ALD}: FAST$$

$$v_{EN}: FAST$$

$$v_{FBP1}: FAST$$

$$v_{GAPDH}: FAST$$

$$v_{GK} = \frac{V_{max,GK}[Glc]}{[Glc] + 15.9}\left(1 - \frac{0.75 \cdot [Fru6P]}{[Fru6P] + 0.005}\right)\frac{[ATP][Glc]}{([ATP] + 0.55)([Glc] + 9)}$$

$$v_{GlcT_{ER}}: FAST$$

$$v_{GLUT2} = 90.9 \cdot \left([Glc_{ext}] - \frac{[Glc]}{1 + \frac{[Glc_{ex}]}{17.3} + \frac{[Glc]}{17.3}}\right)$$

$$v_{GP} = \frac{V_{max,GP}[Glyc]}{300} \cdot \frac{0.265 \cdot [AMP]}{[AMP] + 0.017} \cdot \frac{[Glyc][P] - \frac{[Glc1P]}{0.21}}{\left(1 + \frac{Gly}{1.8}\right)\left(1 + \frac{[P]}{2.2}\right) + \left(1 + \frac{[Glc1P]}{0.7}\right) - 1}$$

$$v_{G6P_{ER}} = \frac{V_{max,G6P_{ER}}[Glc6P_{ER}]}{[Glc6P_{ER}] + 1.84}$$

$v_{GPI}: FAST$

$v_{G1PI}: FAST$

$v_{G6PT_{ER}}: FAST$

$$v_{GS} = \frac{V_{max,GS}(300 - [Glyc])}{300 - [Glyc] + 0.1 \cdot 300} \cdot \frac{[UDP - Glc]}{[UDP - Glc] + 32\left(1 - \frac{[Glc6P]}{[Glc6P] + 0.09}\right) + 0.3}$$

$$v_{LacT} = \frac{5.83 \cdot 10^2 \cdot ([Lac_{ext}] - [Lac])}{1 + \frac{[Lac]}{2.42} + \frac{[Lac_{ext}]}{2.42}}$$

$v_{LDH}: FAST$

$v_{MalT}: FAST$

$v_{MDH}: FAST$

$v_{MDH_{mito}}: FAST$

$$v_{PC} = \frac{3.59 \cdot 10^2 \cdot [ATP_{mito}][Pyr_{mito}][CO2_{mito}] - \frac{[OA_{mito}][ADP_{mito}][P_{mito}]}{6.55}}{([ATP_{mito}] + 0.14)([Pyr_{mito}] + 0.33)([CO2_{mito}] + 4.2)}$$

$$v_{PEPCK} = \frac{V_{max,PEPCK}\left([OA][GTP] - \frac{[PEP][GDP][CO2]}{110}\right)}{\left(1 + \frac{[OA]}{0.024}\right)\left(1 + \frac{[GTP]}{0.021}\right) + \left(1 + \frac{[PEP]}{0.4}\right)\left(1 + \frac{[GDP]}{0.02}\right)\left(1 + \frac{[CO2]}{1.194}\right) - 1}$$

$v_{PEPCK_{mito}}: FAST$

$v_{PEPT}: FAST$

$v_{PFK1}: FAST$

$$v_{PFK2} = \frac{V_{max,PFK2}[Fru6P]}{[Fru6P] + 0.05} \frac{[ATP]}{[ATP] + 0.5}\left(1 - \frac{0.85 \cdot [PEP]}{[PEP] + 0.25}\right)$$

$$v_{FBP2} = \frac{V_{max,FBP2}[Fru26P2]}{([Fru26P2] + 0.002)\left(1 + \frac{[Fru6P]}{0.02}\right)}$$

$v_{PGK}: FAST$

$v_{PGM}: FAST$

$$v_{PK} = \frac{V_{max,PK}[PEP][ADP]}{[PEP] + 5.8 \cdot \left(1 + \frac{[ATP]}{0.32}\right)\left(1 - \frac{[Fru16P2]}{[Fru16P2] + 0.0095}\right)([ADP] + 0.33)}$$

$v_{PyrMalT}: FAST$

$v_{PyrT}: FAST$

$v_{TPI}: FAST$

$$v_{UGP} = \frac{V_{max,UGP} \cdot \left([UTP][Glc1P] - \frac{[UDP - Glc][PP]}{0.31}\right)}{\left(1 + \frac{[UTP]}{0.2}\right)\left(1 + \frac{[Glc1P]}{0.055}\right)\left(1 + \frac{[UDP - Glc]}{0.06}\right)\left(1 + \frac{[PP]}{0.084}\right) - 1}$$

$$v_{UG6D} = \frac{50 \cdot ([UDP - Glc] - [UDP - GA])}{([UDP - Glc] + 1)([UDP - GA] + 1)}$$
